# Prevalence and Antimicrobial Resistance of *Enterococcus* Species: A Retrospective Cohort Study in Italy

**DOI:** 10.3390/antibiotics10121552

**Published:** 2021-12-19

**Authors:** Mariarosaria Boccella, Biagio Santella, Pasquale Pagliano, Anna De Filippis, Vincenzo Casolaro, Massimiliano Galdiero, Anna Borrelli, Mario Capunzo, Giovanni Boccia, Gianluigi Franci

**Affiliations:** 1 Department of Laboratory and Infectious Disease Sciences, Agostino Gemelli University Hospital IRCCS, 00168 Rome, Italy; maryboccy@gmail.com; 2 Section of Microbiology and Virology, University Hospital “Luigi Vanvitelli”, 80138 Naples, Italy; bi.santella@gmail.com (B.S.); massimiliano.galdiero@unicampania.it (M.G.); 3 Department of Medicine, Surgery and Dentistry “Scuola Medica Salernitana”, University of Salerno, 84081 Baronissi, Italy; ppagliano@unisa.it (P.P.); vcasolaro@unisa.it (V.C.); mcapunzo@unisa.it (M.C.); gboccia@unisa.it (G.B.); 4 Department of Experimental Medicine, University of Campania “Luigi Vanvitelli”, 80138 Naples, Italy; anna.defilippis@unicampania.it; 5 Azienda Ospedaliero Universitaria San Giovanni di Dio e Ruggi D’Aragona, 84131 Salerno, Italy; anna.borrelli@sangiovannieruggi.it; 6 Dai Dipartimento Di Igiene Sanitaria e Medicina Valutativa U.O.C. Patologia Clinica E Microbiologica, A.O.U. San Giovanni di Dio e Ruggi D’Aragona Scuola Medica Salernitana, Largo Città di Ippocrate, 84131 Salerno, Italy

**Keywords:** antimicrobial sensitivity, *Enterococcus* spp., microbial infections, empiric therapy

## Abstract

Antimicrobial resistance represents one of the main threats to healthy ecosystems. In recent years, among the multidrug-resistant microorganisms responsible for nosocomial infections, the *Enterococcus* species have received much attention. Indeed, *Enterococcus* have peculiar skills in their ability to acquire resistance genes and to cause severe diseases, such as endocarditis. This study showed the prevalence and antimicrobial resistance rate of *Enterococcus* spp. isolated from clinical samples, from January 2015 to December 2019 at the University Hospital “San Giovanni di Dio e Ruggi d’Aragona” in Salerno, Italy. A total of 3236 isolates of *Enterococcus* *faecalis* (82.2%) and *Enterococcus* *faecium* (17.8%) were collected from urine cultures, blood cultures, catheters, respiratory tract, and other samples. Bacterial identification and antibiotic susceptibility were performed with VITEK 2. *E. faecium* showed a high resistance rate against ampicillin (84.5%), ampicillin/sulbactam (82.7%), and imipenem (86.7%), while *E. faecalis* showed the highest resistance rate against gentamicin and streptomycin high level, but both were highly sensitive to such antibiotics as tigecycline and vancomycin. Studies of surveillance are an important tool to detect changes in the resistance profiles of the main pathogens. These antimicrobial susceptibility patterns are necessary to improve the empirical treatment guideline of infections.

## 1. Introduction

Antimicrobial resistance (AMR) is among the main threats to health systems globally [1]. A report from the United Nations International Committee highlights that the infections caused by multidrug-resistant microorganisms (MDRs) could cause 10 million deaths by 2050, more than today’s cancer deaths [2]. Furthermore, it has been estimated that MDR infections could lead to health care costs of up to $20,000 per patient, leading to a possible global health economic crisis [3]. Moreover, the antimicrobial agents used for the treatment of these infections are very often expensive and toxic, with a limited efficiency over time, leading to an increase in the mortality rate [4,5]. One of the main causes of the development of AMR has been the improper and excessive use of antibiotics. It is assumed that the resistance rate to antibiotics will spread not only in hospital settings but also in communities [6]. The use and abuse of antibiotics by factory farms is contributing to the rapid global growth and spread of AMR [7]. In recent years, among the MDR bacteria responsible for nosocomial infections, the *Enterococcus* spp. received particular attention due to its ability to cause urinary tract infection, bacteremia, and infective endocarditis [8,9]. Enterococci are Gram-positive, opportunistic microorganisms that normally inhabit the human gastrointestinal tract [10]. Among *Enterococcus* spp., *E. faecium* and *E. faecalis* are the third leading cause of nosocomial infections after *S. aureus* and *P. aeruginosa* [11]. It has been estimated that *E. faecium* is responsible for 5–10% of these infections, while those caused by *E. faecalis* are 85–90% [12]. In recent years, infections caused by Enterococci have raised particular concern due to their ability to acquire resistance against many antimicrobial drugs used in clinical practice and to establish severe life-threatening infections in patients living with chronic diseases or cancer [13,14,15]. We previously reported evidence of a high resistance rate to aminoglycosides, Lincosamides and Streptogramins, by these microorganisms [16]. Furthermore, the emergence of Vancomycin-resistant *Enterococcus* (VRE) has increased the use of alternative antibiotics, such as daptomycin and linezolid, towards which increasing resistance trends are developing [17,18,19]. To counter this phenomenon, epidemiological surveillance studies are needed to improve the clinical use of antibiotics and reduce the excessive and improper use of antibiotics [20].

The aim of this study was to evaluate the rates of antimicrobial resistance of *E. faecium* and *E. faecalis*, isolated from clinical samples from 2015 to 2019 in University Hospital, in order to identify suitable drugs for improving empirical therapy.

## 2. Results

A total of 3236 strains of *Enterococcus* spp. isolated from clinical samples were analyzed from January 2015 to December 2019 at the University Hospital “San Giovanni di Dio e Ruggi d’Aragona” in Salerno. Among the patients included in this study, there were 1900 females (58.7%) and 1336 males (41.3%) (Table 1).

The most isolated species was *E. faecalis* (82.2%) compared to *E. faecium* (17.8%). These species were isolated from different samples: urine cultures (32.5%), wound swabs (15.9%), vaginal swabs (19.4%), blood cultures (8.2%), catheters (3.9%), sputum and BAL samples (2.6%), sperm cultures (0.6%), and others (16.9%) (Table 2).

*Enterococcus faecalis* showed a high rate of resistance to gentamicin and streptomycin high level, while it was highly sensitive to ampicillin (96.7%), ampicillin/sulbactam (99.4%), imipenem (98.3%), linezolid (99.4%), nitrofurantoin (99.6%), teicoplanin (98.5%), tigecycline (98.9%), and vancomycin (98.2%). Moreover, *E. faecalis* exhibited a lower rate of sensitivity to levofloxacin (65.1%) (Table 3 or Figures S1 and S2).

*Enterococcus faecium*, on other hand, showed a high resistance to ampicillin (84.5%), ampicillin/sulbactam (84.2%), and imipenem (86.7%), while it was highly sensitive to quinupristin/dalfopristin (83.4%), linezolid (99.7%), teicoplanin (96.8%), tigecycline (99.3%), and vancomycin (96.8%). Finally, *E. faecium* showed a lower rate of sensitivity to streptomycin high level (26%) and gentamicin high level (40%) (Table 4 or Figures S3 and S4).

The resistance rate to vancomycin of *E. faecium* was 3.7% in 2017, 1.6% in 2018, and 6.1% in 2019. In this case, there is not a constant increase but just a fluctuation in the resistance rate over the period of study. Finally, in Table 5, the antibiotic resistance values of each species to the antibiotics show a greater resistance of *E. faecium* compared to *E. faecalis* isolates.

## 3. Discussion

This study was conducted at the “San Giovanni di Dio e Ruggi d’Aragona” University Hospital on *Enterococcus* spp. isolates collected from January 2015 to December 2019. *Enterococcus* spp. has been of particular concern in recent years due to their ability to spread easily in hospital settings among healthcare workers and hospitalized patients and have been included among the main microorganisms causing nosocomial infections [21,22]. Another major problem associated with infections by *Enterococcus* spp., along with the increased incidence rate, is their increasing resistance to antimicrobial agents [23,24].

In the present study, we found a higher prevalence of *E. faecalis* (82.2%) than *E.* *faecium* (17.8%) isolated from clinical samples. This species distribution is similar to that reported from other studies, such as that by Fawzia et al., in which, among 231 *Enterococcus* species isolated, 168 (72.7%) were identified as *E. faecalis* and 53 (22.8%) as *E. faecium* [17,25,26]. These species were isolated at a higher frequency from urine cultures (32.5%), vaginal swabs (19.4%), and wound swabs (15.9%). In agreement with a similar study, the maximum number of isolates were obtained from urine (46.6%), followed by wound swabs (19.4%) [27]. These data highlight the prevalence of Enterococci in urinary tract infections (UTI). Moreover, we reported a low percentage (3.9%) of species isolated from catheters. Probably, one of the main causes may be due to the ability of the Enterococci species to produce biofilms on medical devices such as catheters, pacemakers, and prosthetic heart valves [28]. Many studies have shown the association between biofilm-producing enterococci and urinary catheters in persistent infections [29,30]. The treatment of UTI involves the use of broad-spectrum antibiotics, which are the main cause of the spread of vancomycin-resistant *Enterococcus* strains (VRE).

All of the isolates in our study showed a high sensitivity to linezolid, teicoplanin, and vancomycin, in accordance with data reported by Gupta et al. [31]. Further, the sensitivity rate to linezolid agreed with studies conducted in India and Bangladesh, which reported the absence of *Enterococcus* spp. isolates resistant to linezolid [32,33]. 

In our study, the frequency of Vancomycin-resistant Enterococci (VRE) was 1.3% in 2017, 1.8% in 2018, and 1.9% in 2019 for *E. faecalis* and 3.7% in 2017, 1.6% in 2018, and 6.1% in 2019 for *E. faecium,* according to European surveillance data. *E. faecium*, in fact, showed a variable VRE incidence between different countries: less than 20% in Finland and the Netherlands and more than 20% in Ireland, Greece, and Portugal, confirming that Italy is a country with a low VRE incidence (4.2%) [34]. On the contrary, the reported incidence of VRE in a single center in Brazil was 15.8% [35]. A higher incidence rate of VRE was reported from Iran (23.7%) and from pediatric haematological / cancer patients in Egypt (75.0%) [36,37].

The rapid spread of VRE has led to the use of new antibiotics such as linezolid, teicoplanin, and tigecycline. Fortunately, this study showed high sensitivity to these antibiotics by *Enterococcus* isolates; also, the rate of resistance to teicoplanin was higher than others, with values similar to vancomycin.

Moreover, a relevant percentage ranging from 7.3 to 9.8% of *Enterococcus* was isolated from blood cultures. In a recent retrospective study in China, the incidence of enterococcal bloodstream infections (BSI) of hospitalized patients was 3.9 episodes per 10,000 admissions, with the major pathogen being *E. faecium* (74%) [38]. However, similar studies reported an incidence of 9–14 episodes per 100,000 in Switzerland and 6.9 episodes per 100,000 in a large Canadian region [39,40]. These reports suggest that nosocomial enterococcal BSI are on the rise and that the overall mortality is quite high, ranging from 25–50% [9,41,42]. Many studies reported that bacteremia caused by VRE strains leads to higher mortality rates (2.5-fold increase) as compared to bacteremia caused by vancomycin sensitive strains [43]. In addition, the tigecycline resistance rates in *E. faecium* and *E. faecalis* were reported as 0.7% and 0.5%, respectively. These data are very important because this drug is used to treat bacteremia caused by MDR enterococci.

Moreover, another relevant aspect is that the highest resistance rate was against gentamicin and was observed in 60% for both *E. faecalis* and *E. faecium.* This finding is different than previously reported by similar investigations, particularly for *E. faecalis*, which reported a lower resistance rate to gentamicin than *E. faecium* [44]. It reflects the characteristics of *Enterococcus* resistance reported in our area by the surveillance systems and has a practical impact on different fields [45]. Aminoglycosides, such as gentamicin, have a synergistic effect in combination with Penicillin or Glycopeptides for the therapy of enterococcal infections [46]. This synergistic effect is lost if the strains exhibit a high level of resistance to Aminoglycosides [47]. In fact, high-level resistance to gentamicin is associated with an increase in terms of mortality, as was observed in a population-based study in Denmark that analyzed a sample of over 1000 patients with an infection sustained by *Enterococcus* [41]. Moreover, the incidence of high-level resistance to gentamicin should be considered in prosthetic surgery, where gentamicin can be used in the biomaterials to reduce the risk of infection [48].

## 4. Materials and Methods

### 4.1. Samples Collection

The data of this study were collected from January 2015 to December 2019 at the University Hospital “San Giovanni di Dio e Ruggi d’Aragona” in Salerno, Italy. Samples from patients aged 1 to 99 years with an infection caused by *E. faecium* and *E. faecalis* were included. The samples included urine culture, wound swab, vaginal swab, blood culture, catheters, sputum, bronchoaspirate, sperm culture, and others (abscess, drainage, ascitic fluid, pleural fluid, culture liquor, and purulent material). The treatment of clinical samples was carried out according to the routine identification guidelines of clinical bacteriology, briefly described below. The samples were immediately transported to the bacteriology laboratory and were processed within one hour of sampling.

### 4.2. Identification and Antimicrobial Susceptibility Testing

For the blood culture sample, a volume of 5–10 mL and 2–3 mL were inoculated in blood culture bottles for adult and pediatric patients, respectively, and were incubated in the automated blood culture monitoring BACTEC 9240 blood culture system (Becton Dickinson Diagnostic Instrument Systems) for up to 5 days (21 days if endocarditis was suspected). Subsequently, one drop from each positive bottle was plated on a standard bacteriological media: Chocolate agar, Blood agar, Columbia agar, McConkey agar, and Sabouraud glucose agar medium (bioMérieux-l’Etoile, Marcy-l’Étoile, France). Only the Chocolate agar plate was maintained in the presence of CO_2_. On the other hand, respiratory tract samples, wound swabs, and other samples were plated directly on standard bacteriology media [49,50]. Lastly, for urine culture, one drop was plated on CPSE chromogen plates (bioMérieux-l’Etoile, Marcy-l’Étoile, France). All plates were incubated at 37 °C for 18–36 h.

The bacterial identification and antimicrobial susceptibility test were performed with Vitek 2 (bioMerieux, Marcy l’Etoile, France) using an identification card (ID-GP) and susceptibility cards (AST-658), according to the manufacturer’s instructions. Each bacterial suspension was prepared from pure cultures of bacteria cultivated on plates. Bacterial cells were suspended in 3 mL of a 0.45% sodium chloride solution. The suspension used in the VITEK 2 system was adjusted to a McFarland standard of 0.5 by using a Densicheck (bioMérieux, Marcy l’Etoile, France). The results of antimicrobial susceptibility were interpreted according to EUCAST guidelines [51]. The Quality Control process encompasses the annual service and certification of the instrument by bioMérieux and the Quality Control for cards was *Enterococcus casseliflavus* ATCC 700327™.

The following antibiotics (bioMerieux, Marcy l’Etoile, France) were included in the present study: Ampicillin (AMP), Ampicillin/Sulbactam (SAM), Cefuroxime (CXM), Gentamicin High Level (GEN), Quinupristin/Dalfopristin (QDA), Imipenem (IPM), Levofloxacin (LVX), Linezolid (LNZ), Nitrofurantoin (FTN), Streptomycin High Level (STH), Teicoplanin (TEC), Tigecycline (TCG), and Vancomycin (VAN).

### 4.3. Statistical Analysis

The demographic data of patients, including age, gender, isolated strain(s), and drug sensitivity results, were used for the analysis. The age- and sex-standardized incidences were calculated. A chi-square test was used to compare the differences in the incidence of bacteria in hospitalized patients and the differences among antibiotic sensitivities over the range of years considered in the study. A statistically significant association was found with the *p*-value < 0.05 and an α equal to 5%. The IBM Statistical Package for Social Sciences Version 22.00 (SPSS Inc., Chicago, IL, USA [http://www.spss.com, (accessed on 16 October 2021)] was used for data analysis.

## 5. Conclusions

In recent years, *Enterococcus* spp. has been of particular concern due to their increased incidence and the scarcity of treatments available to counter them. The aim of our study was to understand not only the incidence of *Enterococcus* spp. in clinical samples but also the pattern of antibiotic resistance in order to help to identify the most effective pharmacological treatments against these microorganisms and therefore limit their spread. The monitoring of antibiotic resistance could provide valuable assistance to clinics in the selection of effective empirical therapy. However, due to the long time required for an antibiogram, the search for efficient empirical therapy becomes necessary. Finally, the studies of surveillance are an important tool that aim to provide the antimicrobial susceptibility patterns information for improving empirical therapy, accelerating time to cure, and reducing hospitalization times. To date, genotyping techniques and molecular investigations, at a reduced cost, could accelerate the identification and indicate the presence of resistance genes in a shorter time. This, on the one hand, could reduce the use of broad-spectrum antibiotics and the spread of MDRs and, on the other hand, give space to genetic epidemiology studies that improve the knowledge of the association between genotypes and resistant phenotypes.

## Figures and Tables

**Table 1:** Characteristics of the study population.

Gender	2015	2016	2017	2018	2019	Total
	% (*n*)	% (*n*)	% (*n*)	% (*n*)	% (*n*)	% (*n*)
**F**	51.5 (118)	57.9 (390)	61.4 (485)	61 (486)	56.4 (421)	58.7 (1900)
**M**	48.5 (111)	42.1 (284)	38.6 (305)	39 (311)	43.6 (325)	41.3 (1336)

**Table 2:** Clinical samples positive for isolates of *Enterococcus* spp.

Samples	2015	2016	2017	2018	2019	Total
	% (*n*)	% (*n*)	% (*n*)	% (*n*)	% (*n*)	% (*n*)
Urine cultures	30.6 (70)	30.4 (205)	35.2 (278)	32.4 (258)	32.4 (242)	32.5 (1053)
Others	22.3 (51)	15.3 (103)	16.5 (130)	17.2 (137)	17.0 (127)	16.9 (548)
Wound swabs	13.1 (30)	14.8 (100)	14.9 (118)	15.8 (126)	19.0 (142)	15.9 (516)
Vaginal swabs	13.1 (30)	22.6 (152)	20.3 (160)	19.8 (158)	17.0 (127)	19.4 (627)
Blood cultures	8.3 (19)	7.1 (48)	7.8 (62)	9.3 (74)	7.8 (58)	8.2 (261)
Catheters	7.0 (16)	6.1 (41)	3.4 (27)	2.3 (18)	3.1 (23)	3.9 (125)
Sputum/bronchoaspirate	3.1(7)	2.5 (17)	1.4 (11)	3.1 (25)	3.4 (25)	2.6 (85)
Sperm cultures	2.6 (6)	1.2 (8)	0.5 (4)	0.1 (1)	0.3 (2)	0.6 (21)

**Table 3:** Resistance rates of the clinical isolates of *Enterococcus faecalis* to antimicrobial agents.

*Enterococcus faecalis*	2015	2016	2017	2018	2019
Antibiotics	R% (*n*)	R% (*n*)	R% (*n*)	R% (*n*)	R% (*n*)
Ampicillin	0.6 (161)	1.7 (525)	0.8 (594)	4.2 (600)	2.3 (575)
Ampicillin/sulbactam	0 (162)	0.3 (509)	0.2 (569)	0.9 (550)	0.4 (540)
Gentamicin High Level	61.1 (162)	63.5 (509)	54.9 (565)	56.7 (522)	60.3 (63)
Imipenem	0 (163)	1.5 (534)	0.8 (595)	3.3 (601)	1.6 (576)
Levofloxacin	34.9 (63)	52.4 (166)	46.3 (203)	38.0 (192)	29.5 (190)
Linezolid	0 (175)	0.4 (528)	0.5 (598)	1.5 (608)	0.9 (581)
Nitrofurantoin	0 (60)	0.6 (166)	0 (202)	1 (191)	0.5 (190)
Streptomycin High Level	51.9 (162)	52.8 (504)	44.3 (566)	45.1 (546)	34.0 (529)
Teicoplanin	1.1 (175)	1.8 (542)	0.8 (601)	1.8 (609)	1.7 (573)
Tigecycline	0 (171)	0.2 (523)	0.2 (596)	1.6 (608)	1.7 (572)
Vancomycin	0.6 (174)	3.1 (541)	1.3 (599)	1.8 (606)	1.9 (575)

**Table 4:** Resistance rates of the clinical isolates of *Enterococcus faecium* to various antimicrobial agents.

*Enterococcus faecium*	2015	2016	2017	2018	2019
Antibiotics	R% (*n*)	R% (*n*)	R% (*n*)	R% (*n*)	R% (*n*)
Ampicillin	81.6 (49)	74.4 (129)	87.8 (189)	91.4 (185)	87.5 (160)
Ampicillin/sulbactam	80.0 (50)	74.4 (129)	85.9 (184)	90.2 (183)	83.3 (156)
Quinupristin/Dalfopristin	2.0 (50)	0.8 (129)	1.1 (183)	0 (26)	0.8 (130)
Gentamicin High Level	60.0 (50)	60.5 (129)	69.6 (184)	70.5 (173)	54.2 (24)
Imipenem	86.0 (50)	81.9 (133)	88.9 (190)	91.4 (186)	88.9 (162)
Linezolid	0 (51)	0 (134)	0 (187)	1.1 (188)	0.6 (166)
Streptomycin High Level	74.0 (50)	73.6 (129)	64.7 (184)	61.7 (183)	64.5 (155)
Teicoplanin	3.7 (54)	0 (134)	3.7 (190)	2.1 (188)	6.7 (164)
Tigecycline	0 (53)	0 (124)	0 (177)	0.5 (186)	1.9 (160)
Vancomycin	3.7 (54)	0 (134)	3.7 (189)	1.6 (187)	6.1 (164)

**Table 5:** Difference in the resistance rates among *E. faecalis* and *E. faecium*.

Antibiotics	*E. faecalis*	*E. faecium*
Ampicillin	1.9	84.5
Ampicillin/sulbactam	0.4	82.8
Gentamicin High Level	59.3	63.0
Imipenem	1.4	86.7
Linezolid	0.7	0.3
Streptomycin High Level	45.6	67.7
Teicoplanin	1.4	3.2
Tigecycline	0.7	0.5
Vancomycin	1.7	3

## Data Availability

Epidemiological data used to support the results of this study are included in the article.

## Supplementary Materials

The following are available online at https://www.mdpi.com/article/10.3390/antibiotics10121552/s1, Figures S1 and S2: Resistance rates of the clinical isolates of *Enterococcus faecalis* to antimicrobial agents, Figures S3 and S4: Resistance rates of the clinical isolates of *Enterococcus faecium* to antimicrobial agents.
