# Supplementary material for: Prevalence and Antimicrobial Resistance of Enterococcus Species: A Retrospective Cohort Study in Italy
Source: Antibiotics (Basel). 2021 Dec 19;10(12):1552. doi: 10.3390/antibiotics10121552 (PMC8698357; doi:10.3390/antibiotics10121552)
Supplement: Supplementary file 1 [file antibiotics-10-01552-s001.zip › antibiotics-1459143-supplementary.pdf]

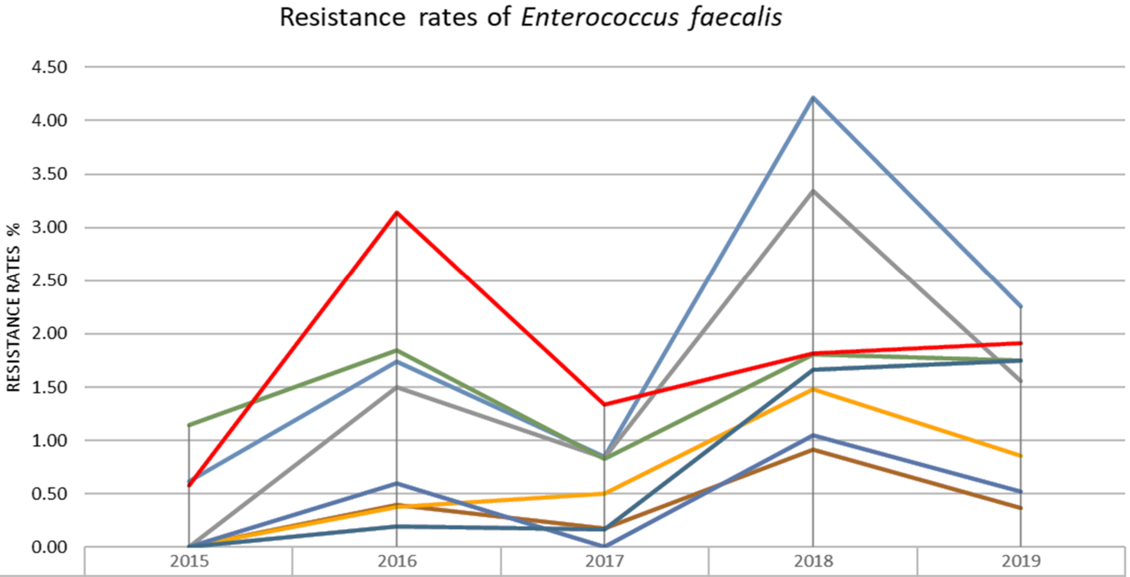

	2015	2016	2017	2018	2019
Ampicillin	0.62	1.74	0.85	4.22	2.26
Ampicillin/sulbactam	0.00	0.39	0.18	0.91	0.37
Imipenem	0.00	1.50	0.84	3.34	1.56
Linezolid	0.00	0.38	0.50	1.48	0.86
Nitrofurantoin	0.00	0.60	0.00	1.05	0.53
Teicoplanin	1.14	1.85	0.83	1.81	1.75
Tigecycline	0.00	0.19	0.17	1.66	1.75
Vancomycin	0.57	3.14	1.34	1.82	1.91

Figure S1. Resistance rates of the clinical isolates of *Enterococcus faecalis* to antimicrobial agents.

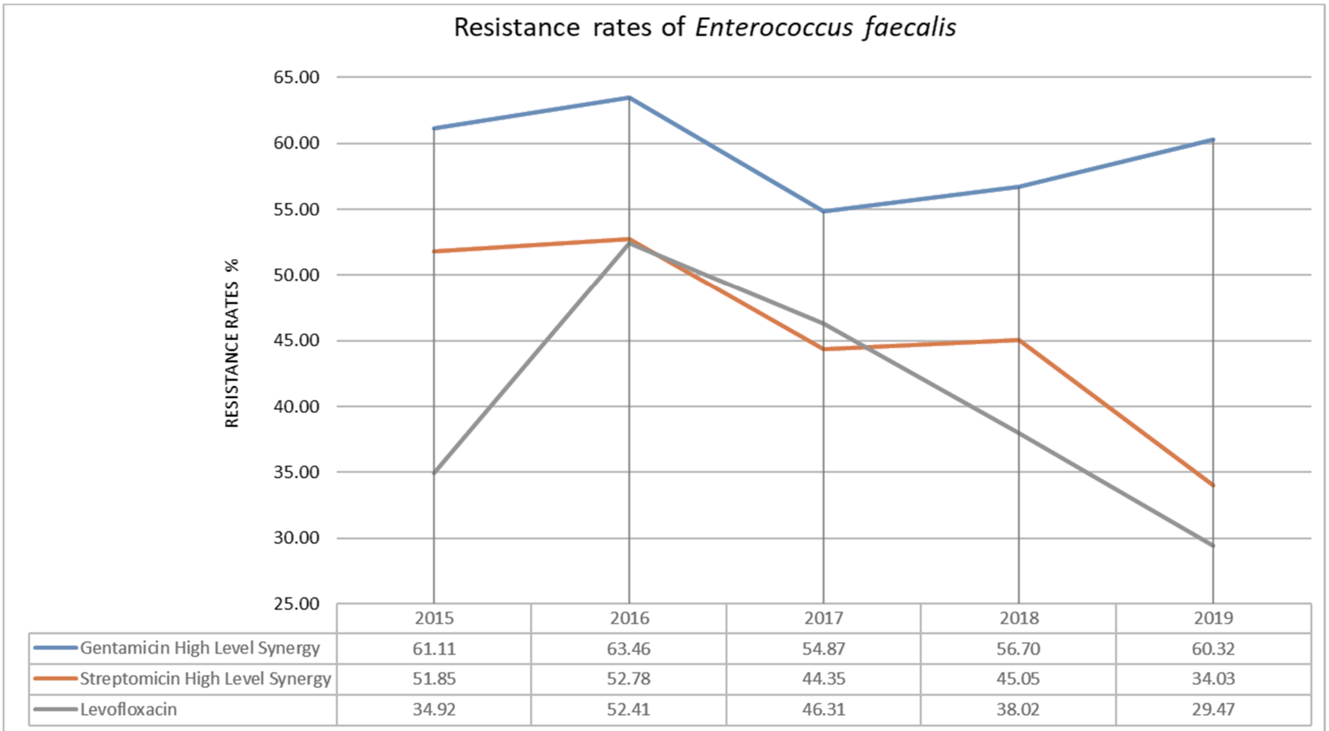

	2015	2016	2017	2018	2019
Gentamicin High Level Synergy	61.11	63.46	54.87	56.70	60.32
Streptomicin High Level Synergy	51.85	52.78	44.35	45.05	34.03
Levofloxacin	34.92	52.41	46.31	38.02	29.47

Figure S2. Resistance rates of the clinical isolates of *Enterococcus faecalis* to antimicrobial agents.

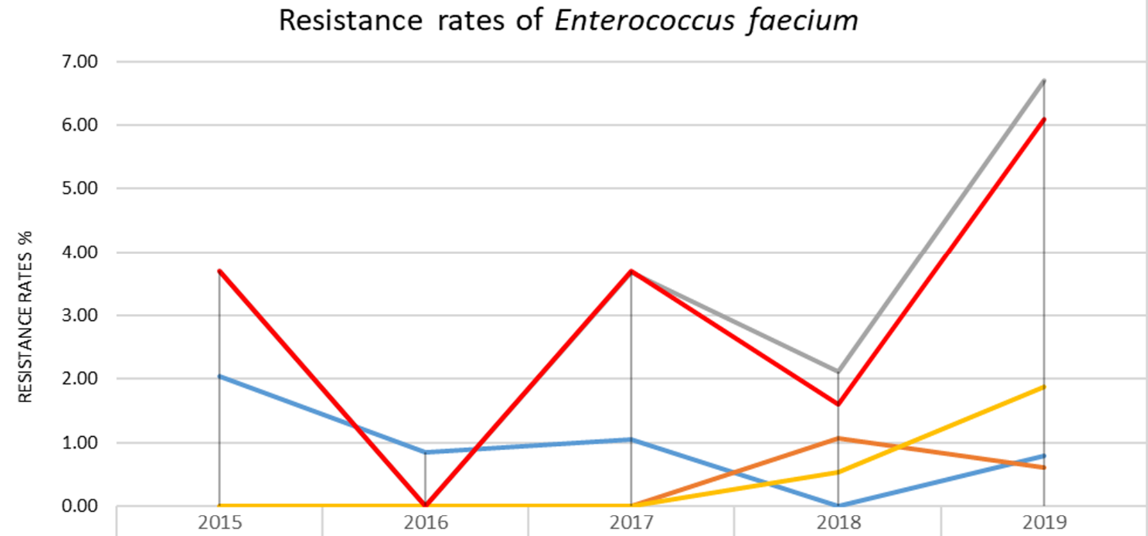

	2015	2016	2017	2018	2019
Chinupristin / Dalfopr	2.04	0.84	1.06	0.00	0.79
Linezolid	0.00	0.00	0.00	1.06	0.60
Teicoplanin	3.70	0.00	3.68	2.13	6.71
Tigecycline	0.00	0.00	0.00	0.54	1.88
Vancomycin	3.70	0.00	3.70	1.60	6.10

Figure S3. Resistance rates of the clinical isolates of *Enterococcus faecium* to antimicrobial agents.

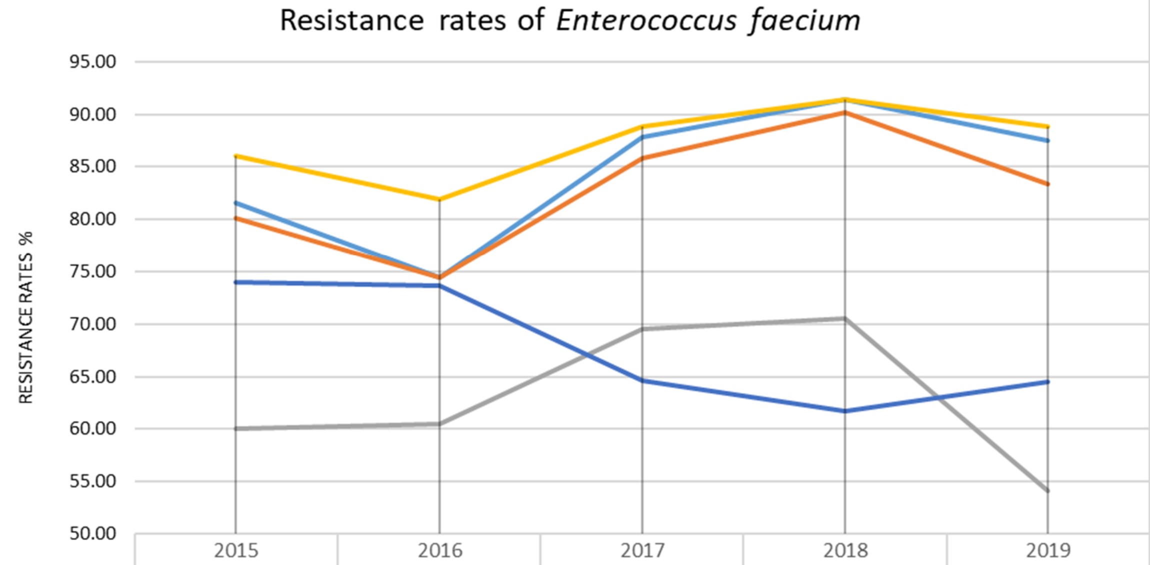

	2015	2016	2017	2018	2019
Ampicillin	81.63	74.41	87.80	91.44	87.51
Ampicillin/sulbactam	80.13	74.43	85.88	90.16	83.33
High level gentamicin	60.00	60.47	69.57	70.52	54.17
Imipenem	86.00	81.89	88.87	91.40	88.89
High level streptomycin	74.00	73.64	64.67	61.74	64.52

Figure S4. Resistance rates of the clinical isolates of *Enterococcus faecium* to antimicrobial agents.
